# Needs for a thorough Joint Clinical Assessment of vaccines within the HTAR: perspectives of NITAGs

**DOI:** 10.1093/eurpub/ckac129.329

**Published:** 2022-10-25

**Authors:** O Wichmann

**Affiliations:** Immunization Unit, Robert Koch Institute, Berlin, Germany

## Abstract

**Issue/problem:**

NITAGs are independent expert advisory committees that provide evidence-based recommendations to the Ministry of Health to guide the introduction of new vaccines and formulate national immunization plans.

**Description of the problem:**

The evaluation of vaccines has become more and more complex. This is not only because the number of vaccines on the market and the diseases they target has increased, but also because a much wider range of population subgroups can be targeted, and for some diseases several vaccines are available and need to be compared. In addition, vaccines not only have an impact on individual well-being, but can also induce population-level effects and broader socioeconomic value.

**Results:**

NITAGs consider to a large extent similar key factors in their evaluation processes. These can be divided into context-free and context-specific aspects. Context-free aspects include effectiveness and safety. Local disease epidemiology, potential vaccine impact at population-level, cost-effectiveness and societal or cultural values and preferences are context-specific aspects. Collaboration is possible when jointly assessing the evidence related to context-free aspects, since this is usually easily transferrable across countries. In 2019, a network of NITAGs in the European Union and Economic Area countries was established under the coordination of the European Centre for Disease Prevention and Control. Within this network, working groups have been established and either performed themselves or supported external contractors in conducting systematic reviews on vaccines effectiveness and safety.

**Lessons:**

The increasing workload of NITAGs and the prospect of joint clinical assessments draw the attention to a number of practical issues to be solved, e.g., consensus on methodological guidelines for systematic reviews, processes to ensure that the products meet NITAGs expectations (content, quality, timing), and approaches how to consider unpublished data.

